# Rod-Like Microglia Are Restricted to Eyes with Laser-Induced Ocular Hypertension but Absent from the Microglial Changes in the Contralateral Untreated Eye

**DOI:** 10.1371/journal.pone.0083733

**Published:** 2013-12-18

**Authors:** Rosa de Hoz, Beatriz I. Gallego, Ana I. Ramírez, Blanca Rojas, Juan J. Salazar, Francisco J. Valiente-Soriano, Marcelino Avilés-Trigueros, Maria P. Villegas-Perez, Manuel Vidal-Sanz, Alberto Triviño, José M. Ramírez

**Affiliations:** 1 Instituto de Investigaciones Oftalmológicas Ramón Castroviejo, Universidad Complutense de Madrid, Madrid, Spain; 2 Facultad de Óptica y Optometría, Universidad Complutense de Madrid, Madrid, Spain; 3 Facultad de Medicina, Universidad Complutense de Madrid, Madrid, Spain; 4 Department of Ophthalmology, School of Medicine, Murcia University, Murcia, Spain; University of Cologne, Germany

## Abstract

In the mouse model of unilateral laser-induced ocular hypertension (OHT) the microglia in both the treated and the normotensive untreated contralateral eye have morphological signs of activation and up-regulation of MHC-II expression in comparison with naïve. In the brain, rod-like microglia align to less-injured neurons in an effort to limit damage. We investigate whether: i) microglial activation is secondary to laser injury or to a higher IOP and; ii) the presence of rod-like microglia is related to OHT. Three groups of mice were used: age-matched control (naïve, n=15); and two lasered: limbal (OHT, n=15); and non-draining portion of the sclera (scleral, n=3). In the lasered animals, treated eyes as well as contralateral eyes were analysed. Retinal whole-mounts were immunostained with antibodies against, Iba-1, NF-200, MHC-II, CD86, CD68 and Ym1. In the scleral group (normal ocular pressure) no microglial signs of activation were found. Similarly to naïve eyes, OHT-eyes and their contralateral eyes had ramified microglia in the nerve-fibre layer related to the blood vessel. However, only eyes with OHT had rod-like microglia that aligned end-to-end, coupling to form trains of multiple cells running parallel to axons in the retinal surface. Rod-like microglia were CD68+ and were related to retinal ganglion cells (RGCs) showing signs of degeneration (NF-200+RGCs). Although MHC-II expression was up-regulated in the microglia of the NFL both in OHT-eyes and their contralateral eyes, no expression of CD86 and Ym1 was detected in ramified or in rod-like microglia. After 15 days of unilateral lasering of the limbal and the non-draining portion of the sclera, activated microglia was restricted to OHT-eyes and their contralateral eyes. However, rod-like microglia were restricted to eyes with OHT and degenerated NF-200+RGCs and were absent from their contralateral eyes. Thus, rod-like microglia seem be related to the neurodegeneration associated with HTO.

## Introduction

Glaucoma is a neurodegenerative disease characterized by axonal degeneration that ends with the death of retinal ganglion cells (RGC) [1-4]. Ocular hypertension (OHT) is the major risk factor for glaucoma, [5-7]. Currently, glaucoma is considered an age-related neurodegeneration and, despite differing in aetiologies, they have many important elements in common, including compartmentalized programs of degeneration targeting axons, dendrites, and finally cell bodies [8].

A close spatial relationship has been established between microglia and multiple retinal ganglion cell (RGC) compartments prior to onset of neurodegeneration [9].. Microglia cells are considered the immunocompetent cells of the CNS. Microglia typically respond to cell damage and degeneration with migration, proliferation, morphological, and immuno-phenotypic changes, as well as production of inflammatory cytokines [10-13]. Indeed, they serve as a neuropathology sensor [14-17] in the brain and are neuroprotective. However, an uncontrolled microglia response may be dangerous to the survival of injured neurons or can even damage healthy neurons afflicted by excessive inflammation [18]. It has also been suggested that cytokines or other factors regulate morphological, as well as functional changes to all three forms of microglia: activated amoeboid, ramified resting microglia, and proliferating rod-like shaped [19]. Rod-like microglial cells are defined by elongated cell bodies with processes that prominently project from the basal and apical ends. It has been hypothesised that these cells participate in neuronal circuit reorganization [20,21]. It is therefore plausible to suggest that rod-like microglia align to either less-injured neurons or axotomized axons in an effort to limit damage. Alternatively, trains of rod-like microglia could just as easily protect uninjured axons [22]. These activated microglia can degrade the extracellular matrix, promote the retraction of dystrophic axons and destabilize synapses [21,23] in a phenomenon called “synaptic stripping” [24,25]. In glaucomatous disease, RGC synapse elimination is involved early in disease progression [26,27]. In addition, in glaucoma, microglia proliferation occurs together with the upregulation of various inflammatory molecules including TNF-alpha and TGF-beta1 [28].

It is widely accepted that activated microglia exert dual functions, which are pro-inflammatory and anti-inflammatory functions. The status of these cells is probably on a continuum between these two extreme states [29]. Unilateral injury to one eye may lead to a microglial response in the eye contralateral to the lesion [30,31]. Previously, our group has studied the qualitative and quantitative changes as well as up-regulation of MHC-II expression on the retinal macroglia and microglia in the contralateral eye of adult Swiss mice after 15 days of unilateral laser-induced OHT [32]. Such changes appeared to take place without a decrease in RGC number [33] or presence of NF-200+RGCs (sign of RGC degeneration) [32]. 

The aim of the present work was to analyse in a model of unilateral laser-induce OHT if: i) microglial activation is secondary to the laser injury or to the rise in IOP; ii) activated microglial cells serve different functions, helping to damage or preserve the RGC in OHT-eyes and contralateral eyes, respectively; and iii) rod-like microglial cells were restricted to eyes with OHT and degenerated NF-200+RGCs and/but absent from their contralateral eyes. 

## Materials and Methods

### Ethics Statement

Mice were treated in accordance with the Spanish Laws and the Guidelines for Humane Endpoints for Animals Used in Biomedical Research. This study was approved by the Ethics Committee for Animal Research of the Murcia University and the Animal Health Service of the Murcia Regional Ministry of Agriculture and Water; approval ID numbers: A1310110807. Also, animal manipulations followed institutional guidelines, European Union regulations for the use of animals in research, and the ARVO (Association for Research in Vision and Ophthalmology) statement for the use of animals in ophthalmic and vision research. 

### Animals and anaesthetics

The experiments were performed on adult male albino Swiss mice (40-45 g) obtained from the breeding colony of the University of Murcia (Murcia, Spain). The animals were housed in temperature- and light-controlled rooms with a 12-h light/dark cycle and *ad libitum* access to food and water. Light intensity within the cages ranged from 9 to 24 lux. Animal manipulation followed institutional guidelines, European Union regulations for the use of animals in research, and the ARVO (Association for Research in Vision and Ophthalmology) statement for the use of animals in ophthalmic and vision research, and was approved by the ethical committee of Murcia University. All surgical procedures were performed under general anaesthesia induced with an intraperitoneal (i.p.) injection of a mixture of Ketamine (75 mg/kg, Ketolar^®^, Parke-Davies, S.L., Barcelona, Spain) and Xylazine (10 mg/kg, Rompún®, Bayer, S.A., Barcelona, Spain). During recovery from anaesthesia, mice were placed in their cages, and an ointment containing tobramycin (Tobrex^®^; Alcon S.A., Barcelona, Spain) was applied on the cornea to prevent corneal desiccation and infection. Additional measures were taken to minimize discomfort and pain after surgery. The animals were killed with an i.p. overdose of pentobarbital (Dolethal Vetoquinol®, Especialidades Veterinarias, S.A., Alcobendas, Madrid, Spain).

### Experimental groups

Three groups of mice were considered for study: an age-matched control (naïve, n=15) and two lasered groups, depending on the region of the eye treated: limbal (OHT group, n=15) and non-draining portion of the sclera, (scleral,n=3); the two latter were processed 2 weeks after lasering. 

### Laser treatment and IOP measurements

To induce OHT, the left eyes of anaesthetized mice were treated in a single session with a series of diode laser burns (Viridis Ophthalmic following previously described methods that are standard in our laboratories [33,34]. Photocoagulator-532 nm, Quantel Medical, Clermont-Ferrand, France). In brief, the laser beam was directly delivered without any lenses, aimed at the limbal and episcleral veins. 

In addition, to ascertain whether the changes observed in microglia were due to the IOP rise or to the inflammatory effects of laser treatment, we studied a scleral group. In this group the laser beam was directly delivered without any lenses, aimed at the non-draining portion of the sclera, specifically 1.2 mm posterior to the limbus of the left eyes to avoid the aqueous-collecting system. In both the limbal- and scleral-treated animal, the spot size, duration, and power were 50-100 µm, 0.5 s, and 0.3 W, respectively. Each eye received between 55-76 burns.

The IOP of the mice was measured under deep anaesthesia in both eyes with a rebound tonometer (Tono-Lab, Tiolat, OY, Helsinki, Finland) [35,36] prior to as well as 24-48 h and 1 week after laser treatment for the lasered group and before being killed for the naïve. At each time point, 36 consecutive readings were made for each eye and averaged. To avoid fluctuations of the IOP due to the circadian rhythm in albino Swiss mice [37] due to the elevation of the IOP itself [38], we tested the IOP consistently around the same time, preferentially in the morning and directly after deep anaesthesia in all animals (lasered group and naïve). Moreover, because general anaesthesia lowers the IOP in the mouse, we measured the IOP of the treated eye (OHT-eye) as well as the contralateral intact fellow eye in all the experiments. 

### Immunohistochemistry

The mice were deeply anaesthetized, perfused transcardially through the ascending aorta first with saline and then with 4% paraformaldehyde in 0.1 M phosphate buffer (PB) (pH 7.4). The orientation of each eye was carefully maintained with a suture placed on the superior pole immediately after deep anaesthesia and before perfusion fixation. Moreover, upon dissection of the eye, the insertion of the rectus muscle and the nasal caruncle were used as additional landmarks [39]. The eyes were post-fixed for 2h in the same fixative and kept in sterile 0.1 M PB. Retinas were then dissected and processed as retinal whole-mounts [40]. 

For the analysis of the microglia population of the NFL and for the determination of the relationship between astrocytes and microglia and the expression of MHC class II, retinal whole-mounts from naïve (n=3), OHT-eyes (n=3) and their contralateral eyes (n=3), scleral group (n=3) and their contralateral eyes (n=3), were triple immunostained, as described elsewhere [32], with the following primary antibodies: rabbit anti Iba-1 (Wako, Osaka, Japan) in a 1/500 dilution, chicken anti-GFAP (Millipore, USA) in a 1/100 dilution and anti-mouse MHC class II (I-A/I-E) (eBioscience; San Diego, CA; USA) in a 1/100 dilution. Binding sites of the primary antibodies were visualized with the corresponding secondary antibodies: donkey anti-rabbit Alexa Fluor 594 (Invitrogen, Paisley, UK) diluted 1/800, DyLight 405-conjugated donkey anti-chicken (Jackson Immuno Research, USA) diluted 1/150 and goat anti-mouse Alexa Fluor 488 (Invitrogen, Paisley, UK) diluted 1/150.

Retinas of naïve (n=3), OHT-eye (n=3) and their contralateral eyes (n=3) were double immunostained with anti-Iba1 plus anti-CD68 (which recognizes a single-chain heavily glycosylated protein of 90-110 kD that is expressed on the lysosomal membrane of macrophages) in order to study the expression of this marker on retinal microglia. The working dilutions were 1/500 for rabbit anti Iba-1 (Wako, Osaka, Japan) and 1/40 for CD68 rat anti-mouse (AbD Serotec, Oxford, UK). Binding sites of the primary antibodies were visualized after two days of incubation with the corresponding secondary antibodies: donkey anti-rabbit Alexa Fluor 594 (Invitrogen, Paisley, UK) diluted 1/800 and goat anti-rat Alexa Fluor 488 (Invitrogen, Paisley, UK) diluted 1/150.

Retinas of naïve (n=3), OHT-eye (n=3) and their contralateral eyes (n=3) were double immunostained with anti-Iba1 plus, anti-CD86 (that recognize a co-stimulatory molecule). The working dilutions were 1/500 for rabbit anti-Iba-1 (Wako, Osaka, Japan) and 1/25 for rat anti-mouse CD86 (BD Pharmigen Europe). Binding sites of the primary antibodies were visualized after two days of incubation with the corresponding secondary antibodies: Donkey anti-rabbit Alexa Fluor 594 (Invitrogen, Paisley, UK) diluted 1/800 and Donkey anti-rat Alexa Fluor 488 (Invitrogen, Paisley, UK) diluted 1/300. 

For the study of the expression of Ym1 (which recognizes a protein from the lectin family synthesised and secreted by alternatively activated macrophages during inflammation) on retinal microglia, retinas of naïve (n=3), and OHT-eye, (n=3), and their contralateral eyes (n=3) were double immunostained with anti-MHC class II plus anti-Ym1. The working dilutions were: anti-mouse MHC class II (I-A/I-E) (eBioscience, San Diego, CA, USA) in a 1/100 dilution and rabbit anti-Ym1 (StemCell Technologies, France) in a 1/75 dilution. Binding sites of the primary antibodies were visualized after two days of incubation with the corresponding secondary antibodies: goat anti-mouse Alexa Fluor 488 (Invitrogen, Paisley, UK) diluted 1/150 and donkey anti-rabbit Alexa Fluor 594 (Invitrogen, Paisley, UK) diluted 1/800.To study the relationship between dendrites, axons, and microglia, we double immunostained the retinal whole-mounts of naïve eyes (n=3), OHT-eye (n=3) and their contralateral eyes (n=3), with the following primary antibodies: rabbit anti Iba-1 (Wako, Osaka, Japan) in a 1/500 dilution and mouse anti-NF-200 (a marker of axonal cytoskeleton, that accumulates abnormally in the soma and dendrites of the RGCs after axonal injury) (Sigma St Louis, MO, USA) in a 1/500 dilution. Binding sites of the primary antibodies were visualized after two days of incubation with the corresponding secondary antibodies: donkey anti-rabbit Alexa Fluor 594 (Invitrogen, Paisley, UK) diluted 1/800 and goat anti-mouse Alexa Fluor 405 (Invitrogen, Paisley, UK) diluted 1/100.

In all instances, a negative control was performed to demonstrate that the secondary antibody reacted only with their respective primary antibody. This control was made by eliminating primary antibody and replacing it with antibody diluent. In addition to identifying the contribution of the endogenous fluorescence to the observed label, a tissue sample was incubated in all the buffers and detergents used in the experiment but without antibodies [40]. 

Retinas were analysed and photographed with the ApoTome device (Carl Zeiss, Germany) coupled to a fluorescence microscope (*Zeiss, Axioplan 2 Imaging Microscope*) equipped with appropriate filters for fluorescence-emission spectra of Alexa fluor 488 (Filter set 10, Zeiss), Alexa fluor 594 (Filter set 64, Zeiss) and DyLight 405 (Filter set 49, Zeiss). The ApoTome uses the “structured-illumination” method that enables conventional microscopy to create optical sections through the specimen and thereby improve the contrast and resolution along the optical axis.

### Statistical Analysis

Data for the statistical analysis were introduced and processed in a SPSS 19.0 (comprehensive statistical software; SPSS Inc^©^). Data are shown as mean±SD. Statistical analyses were performed to compare IOP values of the OHT-eyes, the contralateral and naïve eyes.

## Results

### 1: OHT group

#### Laser-induced ocular hypertension

The IOP values of OHT-eyes (29.55±4.44 mmHg) significantly differed from naïve values (16.16±3.11 mmHg; P<0.001, ANOVA with Bonferroni) and contralateral eyes (15.47±1.57 mmHg; P<0.001, ANOVA with Bonferroni). No significant differences were found between contralateral and naïve eyes. 

#### Iba-1 immunostaining

**Figure 1:**
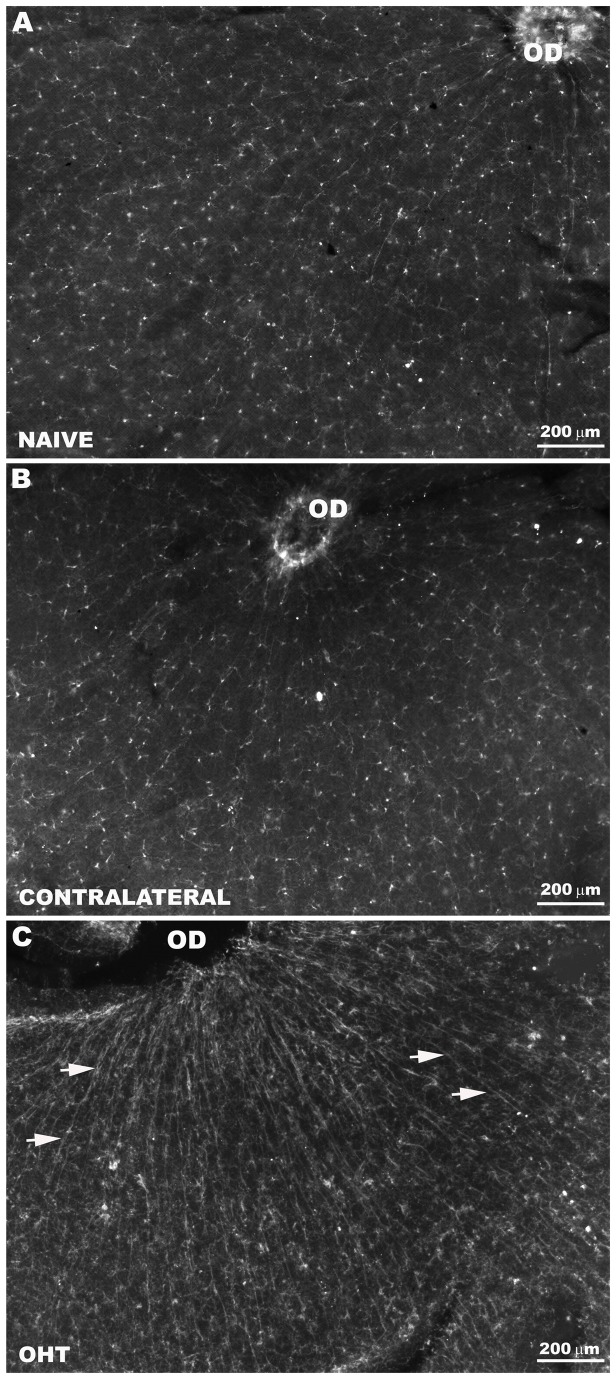
Rod-like microglia were restricted to the NFL of OHT-eyes. Iba-1+ cells in the NFL and RGC layer. Retinal whole-mounts. In naïve (A) and contralateral (B) eyes, Iba-1+ cells were distributed in a mosaic of tiled cells that built networks throughout the entire retina. In OHT-eyes (C), Iba-1+ cells aligned forming parallel trains (arrows) composed by multiple cells exhibiting rod-like morphology. The trains were more evident in the central retina. (NFL: nerve-fibre layer; RGC: retinal ganglion-cell layer; OD: optic disc; OHT: ocular hypertension).

**Figure 2:**
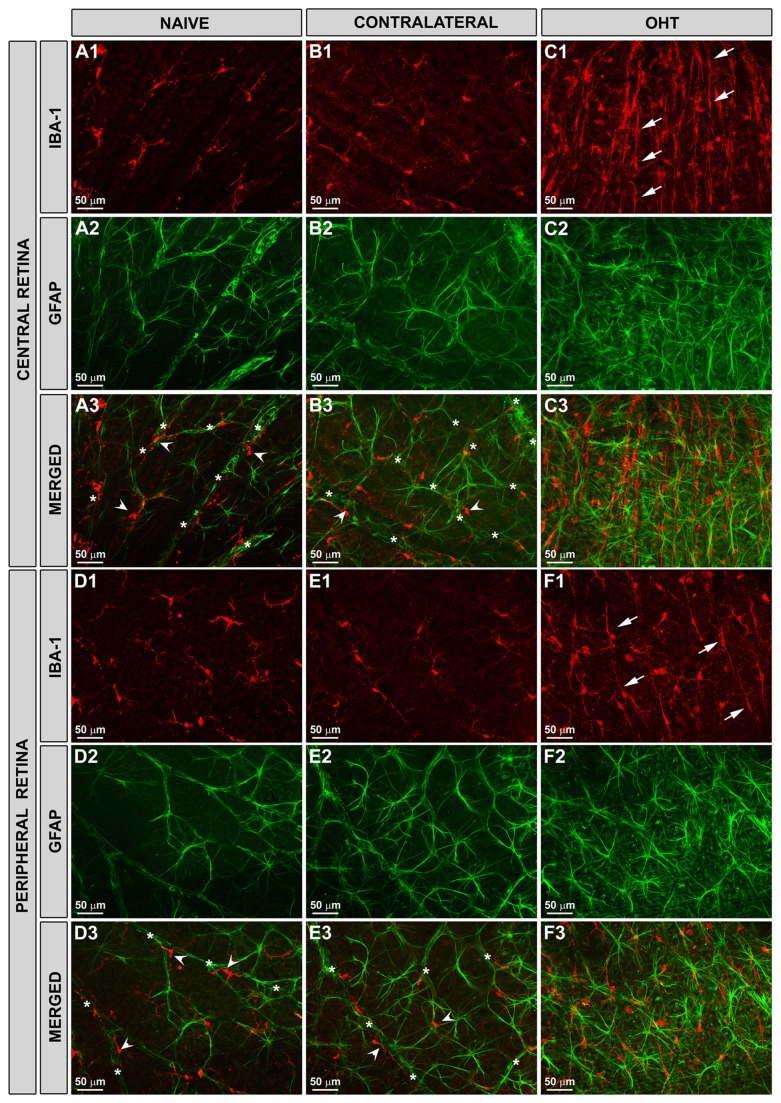
Rod-like microglia did not relate to retinal blood vessels or astrocytes. Iba-1+ and GFAP+ cells in the NFL and RGC layer. Retinal whole-mounts. In naïve (A,D) and contralateral (B,E) eyes, most Iba-1+ cells had a ramified morphology (A1, B1, D1, E1: arrowhead) and their somas were located on or near the vessel walls (A3, B3, D3, E3: asterisks). GFAP+ astrocytes were located among ramified Iba-1+ cells and like these, were related to the vessels (A2-A3, B2-B3, D2-D3, E2-E3). In OHT-eyes (C,F), astrocytes and Müller cells were reactive (C2-C3, F2-F3). No alignment or co-localization of GFAP+ astrocytes with rod-microglia (C1, F1: arrows) was detected. Trains of rod-like microglia were more evident in the central (C1, C3) than in the peripheral retina (F1, F3). (NFL: nerve-fibre layer; RGC: retinal ganglion-cell layer; OHT: ocular hypertension). .

**Figure 3:**
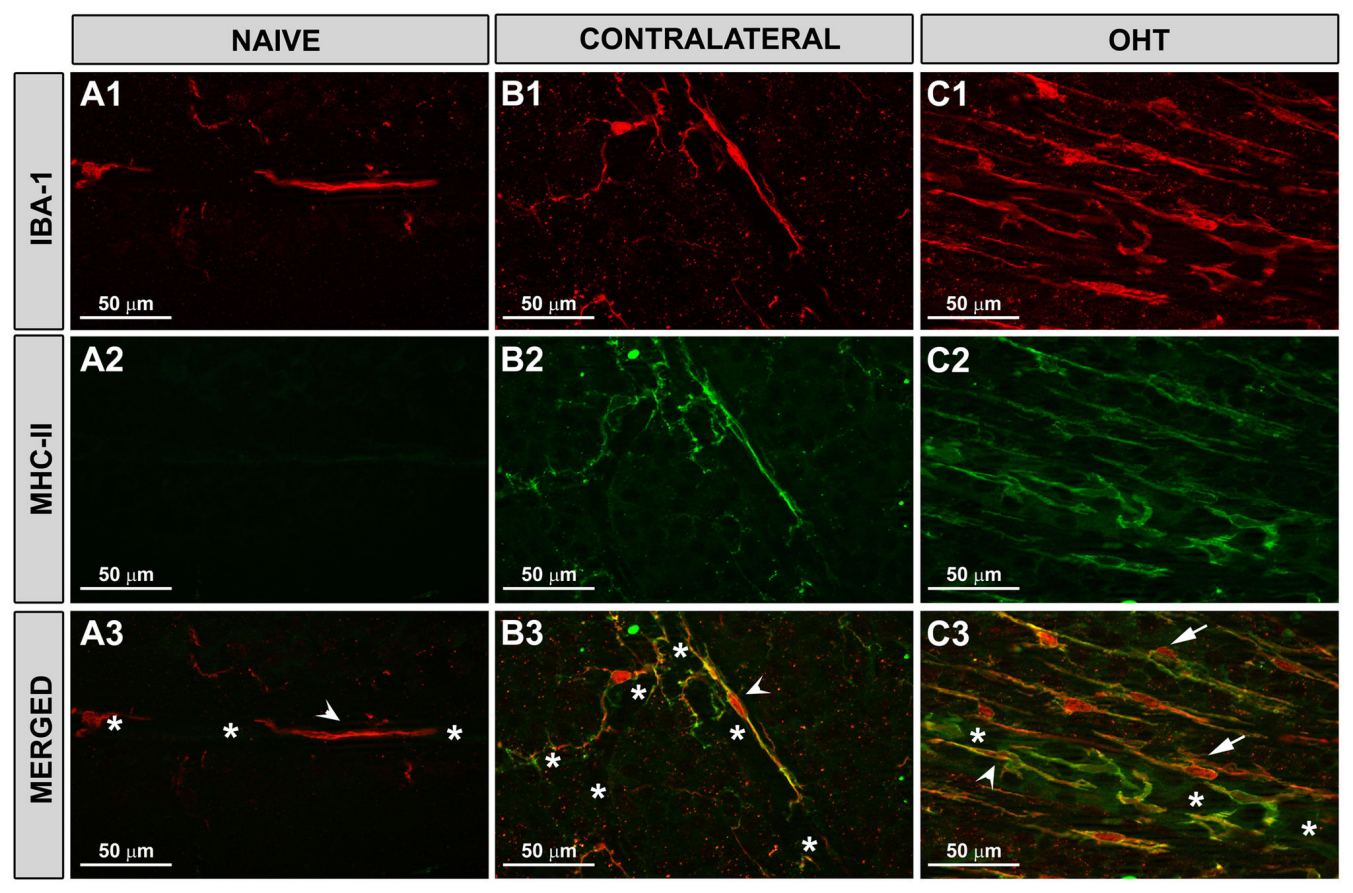
MHC-II was upregulated in contralateral and OHT-eyes. Iba-1+ and MHC-II+ cells in the NFL and RGC layer. Retinal whole-mounts. In naïve (A1-A3), contralateral (B1-B3), and OHT (C1-C3) eyes, some Iba-1+ cells displayed bipolar morphology and perivascular arrangement (arrowhead). These cells were observed mainly in the large retinal vessels (in the vicinity of the optic nerve) and in the collecting tube (in the peripheral retina) and expressed MHC-II. This MHC-II expression, constitutive in naïve eyes (A2-A3), was upregulated in contralateral (B2-B3) and OHT-eyes (C2-C3). (NFL: nerve-fibre layer; RGC: retinal ganglion-cell layer; OHT: ocular hypertension; asterisk: blood vessel; arrow: rod-microglia).

In the naïve and contralateral eyes, Iba-1+ cells were distributed in a mosaic of tiled cells that built networks throughout the entire retina (Figure 1). Most microglia in the NFL exhibited a ramified morphology. They were associated with the retinal vessels and their somas were located on or near the vessel walls (Figure 2). In addition, both in the large retinal vessels (in the vicinity of the optic nerve) and in the collecting tube (in the peripheral retina) of naïve and contralateral eyes, some perivascular Iba-1 positive cells displayed bipolar morphology (Figure 3A,B). Notably, in addition to these two morphological cell types, in the NFL of OHT- eyes, there were Iba-1+cells with a rod-like morphology (elongated cell bodies and two processes prominently projected from each pole) (Figure 2C). These cells did not relate to retinal vessels and aligned end-to-end, coupling to form trains. We use the term “coupling” to indicate a physical proximity between the processes of adjacent rod-like microglia (Figure 1C; Figure 2C1,C3, F1,F3). These trains consisted of multiple cells running parallel to the retinal surface from the optic disc to the periphery. The trains were more evident in the central area than in the intermediate one (Figure 1C; Figure 2C1,C3), and mostly absent in the retinal periphery (Figure 2F1,F3). 

**Figure 4:**
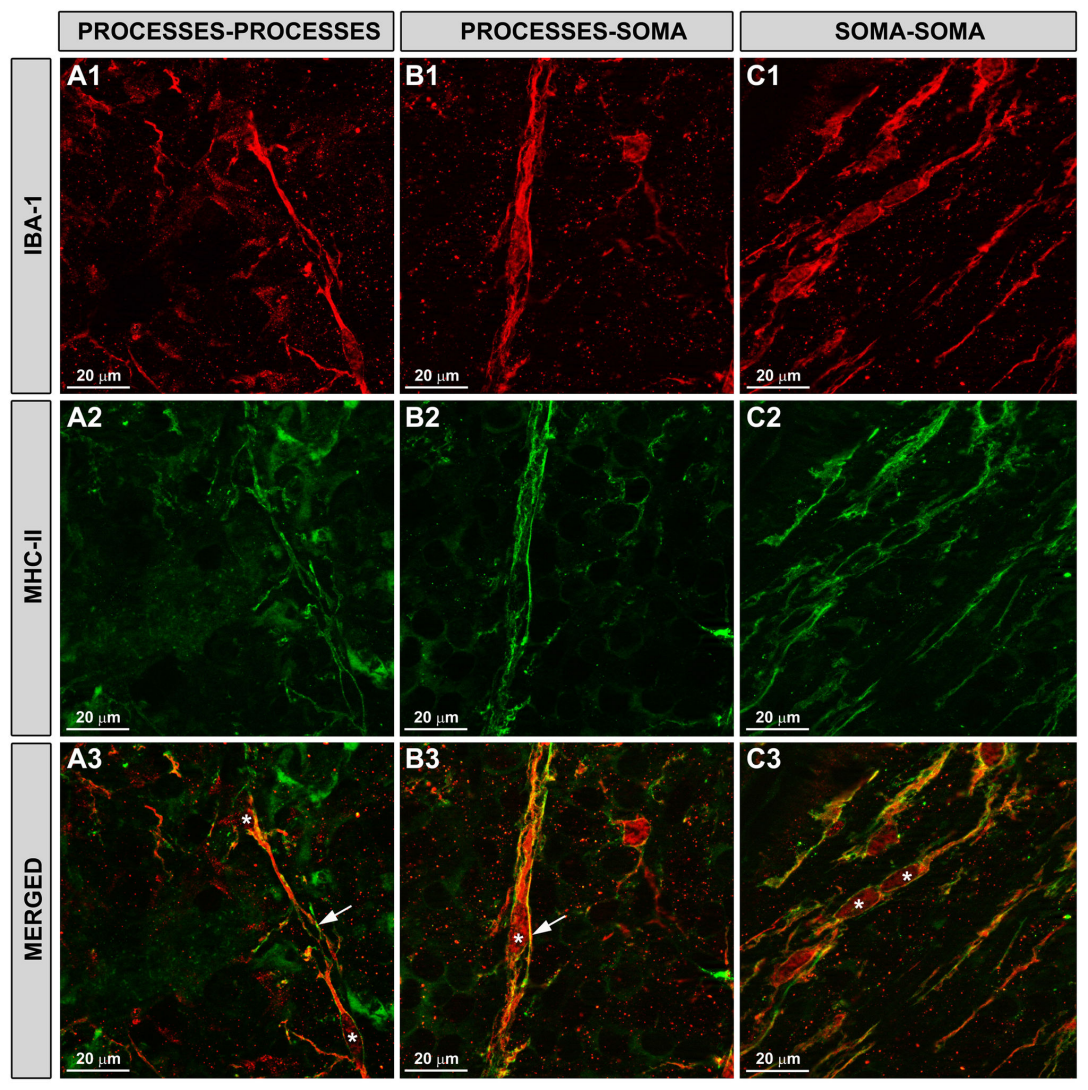
Rod-like microglia expressed MHC-II and related to each other in three different patterns. Iba-1+ and MHC-II+ cells in the NFL and RGC layer. Retinal whole-mounts. Rod-like microglia expressed MHC-II (A2-A3, B2-B3, C2-C3). In the trains, they related to each other in three ways. Process-process (A1-A3): processes of neighbouring rod-microglia in the trains seem to be intertwined; process-soma (B1-B3): the process of one rod-like microglia seems to surround the cell body of the next rod-like microglia in the train; soma-soma (C1-C3): two cells bodies are in apparent close contact. (NFL: nerve-fibre layer; RGC: retinal ganglion-cell layer; OHT: ocular hypertension; asterisk: soma; arrow: processes).

In the trains, rod-like microglia in the NFL of eyes with OHT related to each other in three different ways. First, process to process: the process of one rod-like microglia seemingly intertwined with the process of the next rod-like microglia in the train (Figure 4A). Second, process to cell body: the process of one rod-like microglia seemed to surround the cell body of the next rod-like microglia in the train (Figure 4B). Third, cell body to cell body: the two cell bodies were in apparently close contact (Figure 4C)

**Figure 5:**
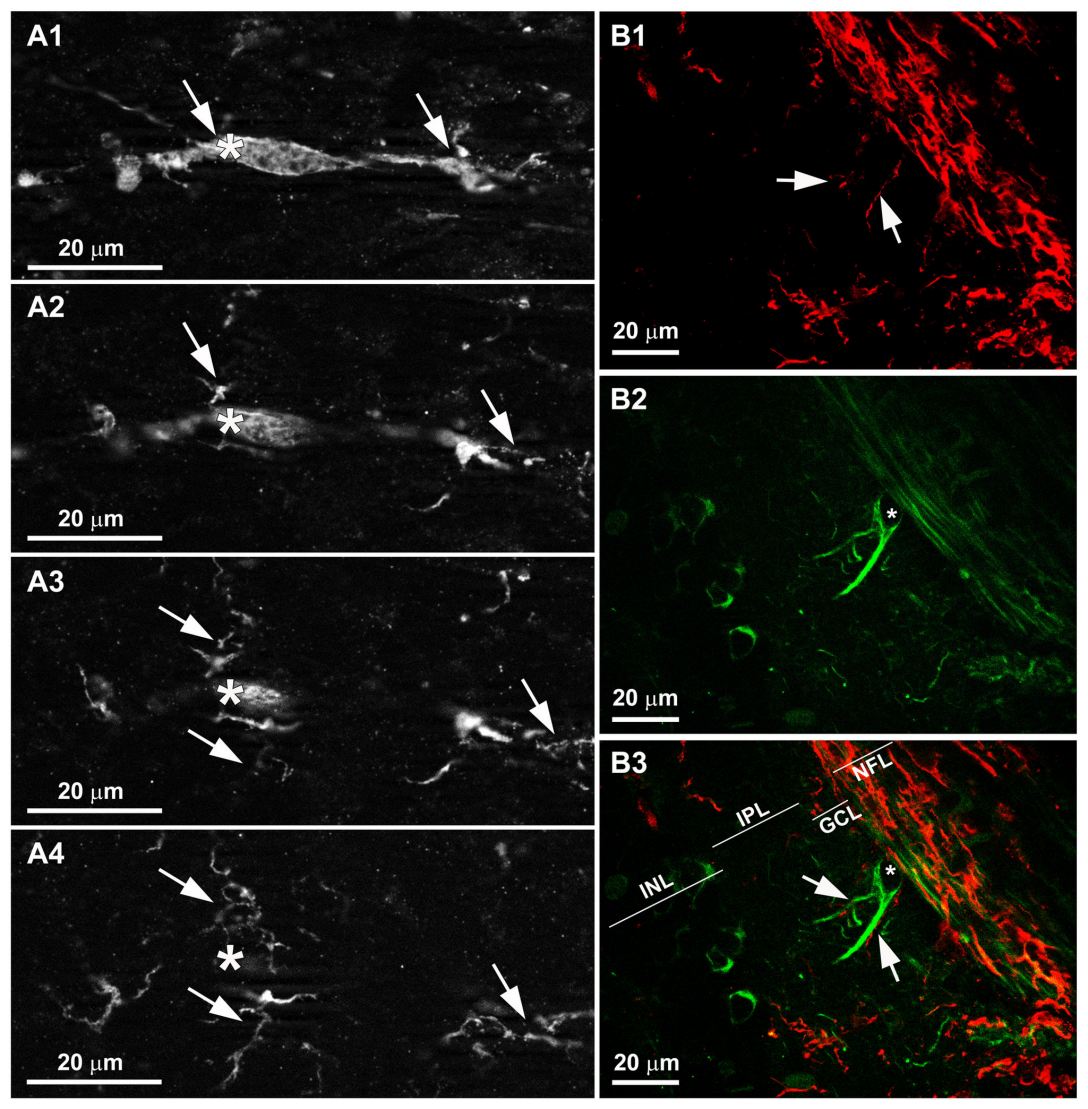
The processes of rod-microglia penetrates the IPL. Iba-1 and NF-200 immunostaining. Retinal whole-mounts. Using the z stack tool associated with the microscope, we observed that some processes of rod-microglia penetrate the ganglion-cell layer and inner plexiform layer (A). The pressure observed by the cover slip on the whole-mount caused retinal-like section effect on one edge of the tissue that revealed that, in the IPL, rod-microglia were apparently in close relation with the dendrites of NF-200+RGCs (B1,B3). (INL: inner plexiform layer; IPL: inner plexiform layer; GCL: ganglion-cell layer; NFL: nerve-fibre layer; RGC: retinal ganglion-cell layer; asterisk: soma; arrow: rod-microglia processes).

Some processes of rod-microglia penetrate the underlying ganglion-cell layer and inner plexiform layer, as observed both using the z-stack tool associated with Zeiss, Axioplan 2 Imaging Microscope as well as on one edge of the tissue due to the retinal-like section effect caused by the pressure exerted by the cover slip on the whole-mount (Figure 5). 

#### Staining of rod-like microglia with markers for activated microglia: MHC-II, CD68, CD86, CD163 and Ym1

**Figure 6:**
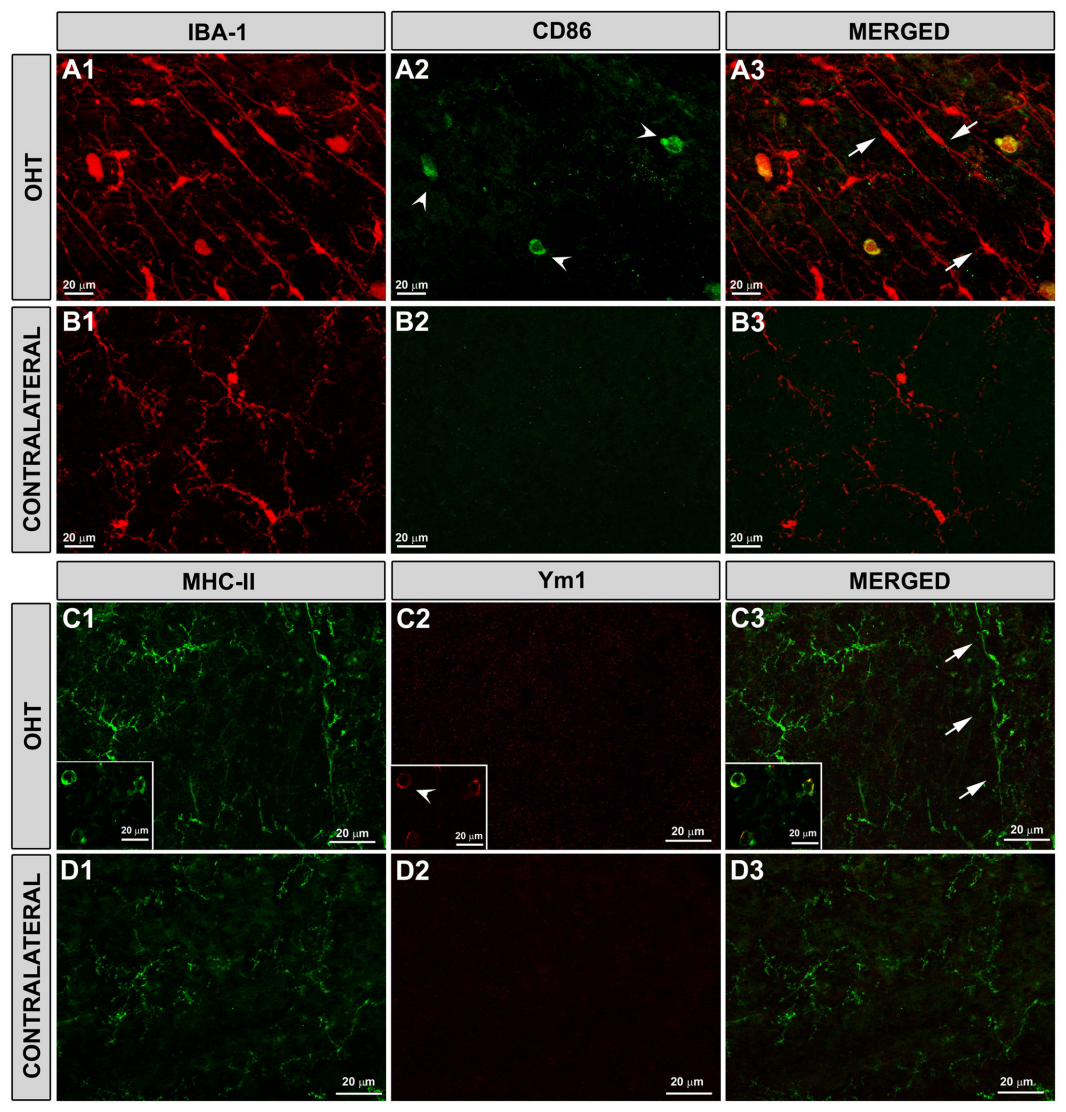
Rod-like microglia did not take either CD86 or Ym1 staining. Iba-1, CD86, MHC-II and Ym1 immunostaining in the NFL. Retinal whole-mounts. In OHT-eyes (A1-A3, C1-C3) CD86 immunostaining (A2, A3) and Ym1 immunostaining (C2-C3: inset) was restricted to few globular cells (arrowhead) located in the NFL and in the vitreal surface of the retina. Rod-like microglia (arrow) did not show either CD86 or Ym1 immunostaining. In contralateral eyes (B1-B3, D1-D3) no CD86+ or Ym1+ cells were detected. (NFL: nerve-fibre layer).

A weak constitutive MHC-II expression was found in some microglial cells in the NFL in naïve eyes (Figure 3A2). By contrast, in contralateral eyes 15 days after laser-induced OHT, Iba-1+ microglia exhibited intense MHC-II immunoreaction (Figure 3B2). In some instances, in the contralateral eyes, some Iba-1+ dystrophic microglia had intense CD68 immunostaining. No CD86 (Figure 6B2) or Ym1 immunostaining (Figure 6D2) was observed in microglial cells in contralateral eyes.

**Figure 7:**
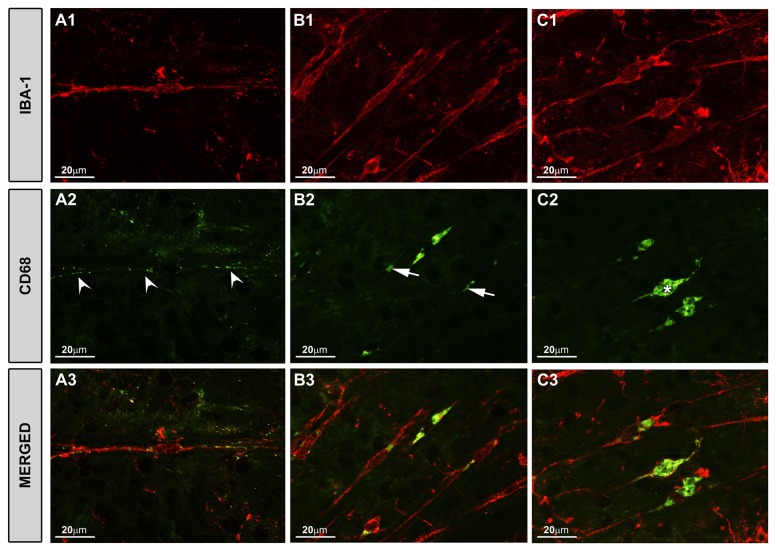
Rod-microglia had different stages of CD68 staining. Iba-1+ and CD68+ cells. Retinal whole-mounts. With respect both to morphological features and to CD68 immunostaining patterns, three stages of rod-like microglia were detected: (A1-A3) rod-like microglia with elongated cell bodies and processes prominently projected from the poles and an intense punctate CD68 staining (arrowhead); (B1-B3) cells with shorter and thicker processes, and coarse patches of CD68 immunoreaction (arrow); (C1-C3) fusiform cell bodies with short and thick bipolar processes and intense CD68 positive cytoplasm (asterisk). In all cases, CD68 immunoreaction was found both in the cytoplasm and processes.

Fifteen days after lasering, rod-like microglia were detected in OHT-eyes. These rod-like microglia had an intense MHC-II+ immunoreaction (Figure 3C2; Figure 4A2,B2,C2) and a punctate CD68 staining throughout the NFL (Figure 7). This staining was evident in rod-like microglia throughout the train, both in the cytoplasm and in the processes. In addition, three stages of rod-like microglia were observed to coexist in OHT eyes. These stages could represent different activation phases considering morphological features as well as CD68 immunostaining patterns: i) an initial stage in which rod-like microglia had elongated cell bodies and processes prominently projected from the poles. These cells had an intense CD68 punctate staining (Figure 7A1-A3); ii) a second stage of cells with shorter and thicker processes and coarse patches of CD68 immunoreaction (Figure 7B1-B3); and iii) a third stage with thick fusiform cell bodies, short and thick bipolar processes and intense CD68 positive cytoplasm (Figure 7C1-C3). In all instances, CD68 immunoreaction was detected both in the cytoplasm and processes.

In the NFL of OHT-eyes no CD86 (Figure 6A1-A3) or Ym1 (Figure 6C1-C3) expression was detected in ramified or rod-like microglia. Positive immunostaining for these markers was found only in scarce globular cells located in the NFL and vitreal surface of the retina (Figure 6A2,C2 inset). 

#### Rod-like microglia, astrocytes, and axons

We performed a double-labelling with Iba-1 and GFAP to study micro- and macroglial cells, respectively. GFAP immunostaining revealed that after 15 days of laser-induced OHT [32] macroglia showed reactivity features and that rod-like microglia and astrocytes were apparently not related to each other (Figure 2).

**Figure 8:**
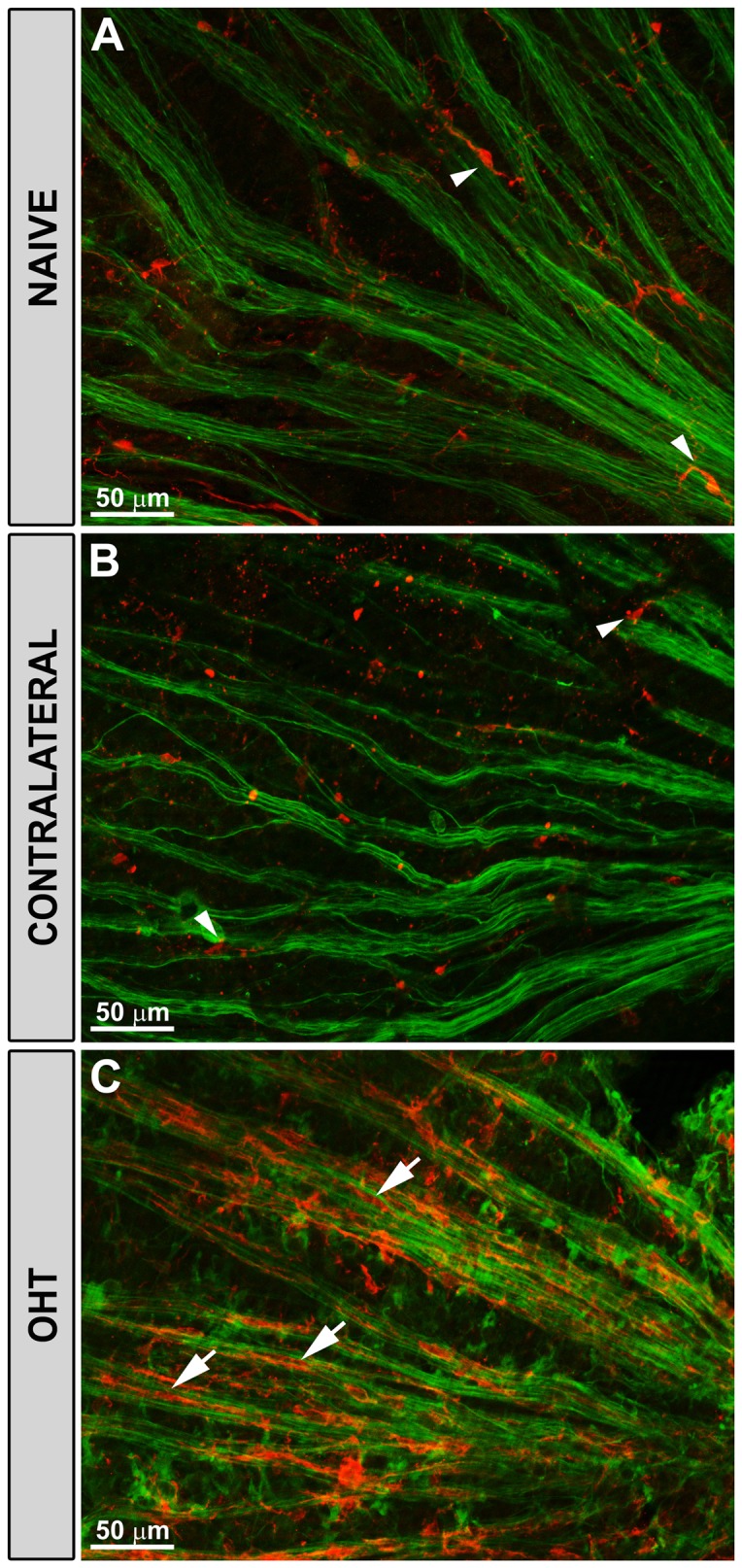
Rod-like microglia was related to axons. Iba-1 and NF-200 immunostaining. Retinal whole-mounts. In naïve (A) and in contralateral eyes (B) the ramified microglia (arrowhead) sent some processes to the axons but did not run parallel to the axons. By contrast, the rod-microglia trains in OHT-eyes (C) run parallel to and close to axons (arrow). (OHT: ocular hypertension).

A double-immunostaining with Iba-1 and NF-200 was performed to study the relationships between rod-like microglia and RCG after axonal injury. In naïve and contralateral eyes RGC axons were uniformly labelled with anti-NF-200 (Figure 8A-B). NF-200+RGC staining was rarely observed in the somas or dendrites of RGCs [32]. In these eyes the ramified microglia were associated with blood vessels (Figure 2A,B,D,E) and sent some processes to the axons (Figure 8A,B). 

**Figure 9:**
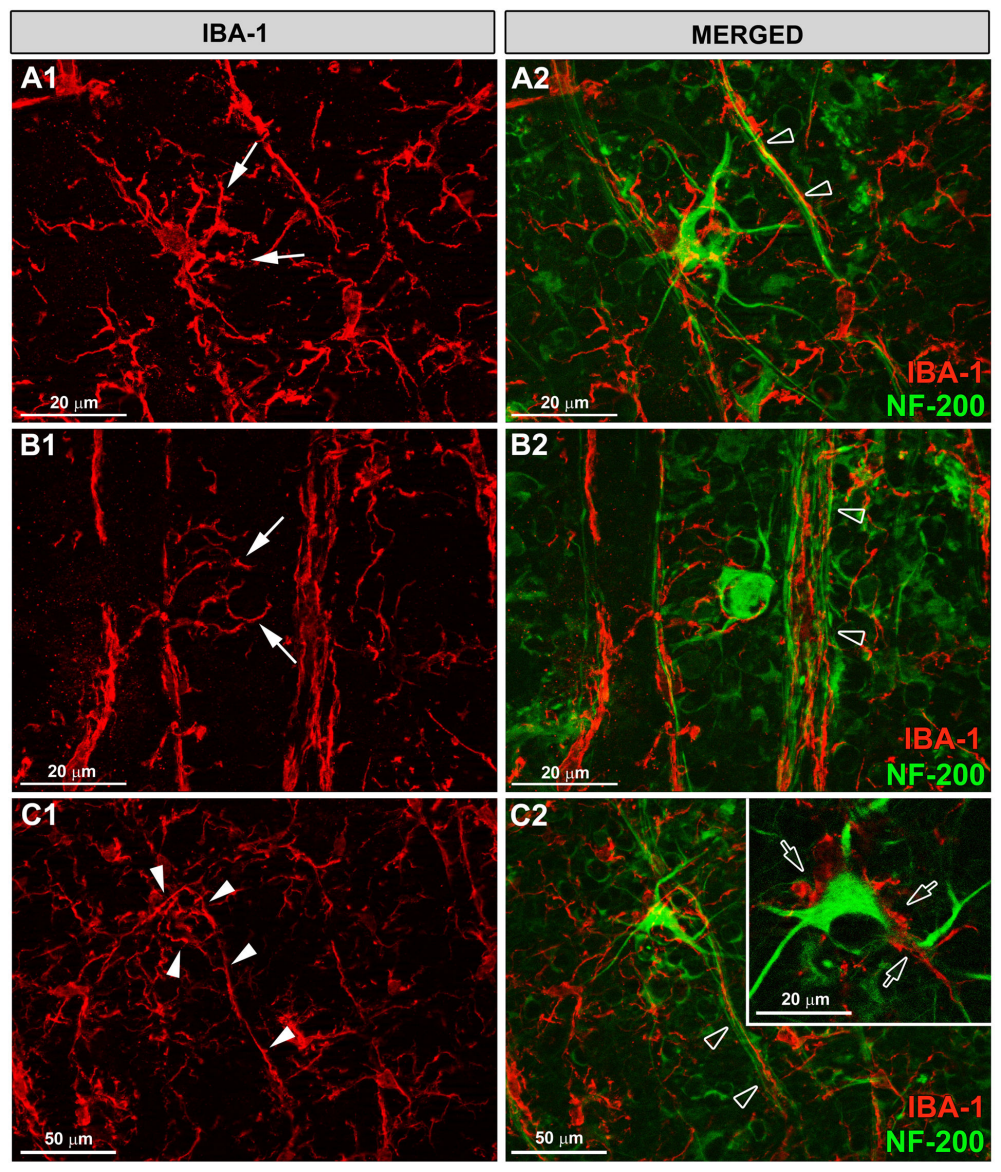
Rod-microglia sent processes to NF-200+RGCs somas and dendrites. Iba-1 and NF-200 immunostaining. Retinal whole-mounts. In OHT-eyes there was an abnormal NF-200 staining of the soma and primary dendrites of some RGCs (A2-C2). Rod-microglial run parallel to and close to axons, with minimal gaps between the two (empty arrowhead). Rod-microglia sent processes to NF-200+RGCs bodies and dendrites (arrows in A1-B2). The processes of the rod-microglia were apparently in close contact with NF-200+RGCs somas and dendrites (empty arrow in inset). In some instances rod-microglia deviates from its straight parallel to the axon and, with its processes, surrounds the soma and proximal dendrites of NF-200+RGCs (arrowhead in C1, C2).

OHT eyes showed an abnormal NF-200 accumulation both in RGC axons as well as in the cell bodies and primary dendrites of some RGCs [32] (Figures 5B2,B3; 9A2,B2,C2). The rod-like microglia trains in OHT eyes were associated with axons (Figure 8C) and not related to retinal vessels (Figure 2C,F). Rod-like microglias were observed parallel to and close to axons, with minimal gaps between the two (Figure 8C; Figure 9A2,B2,C2). Apparently some processes of rod-like microglia were in close contact with NF-200+RGC bodies and dendrites (Figure 5B1-B3; Figure 9). It bears mentioning that in some instances rod-microglia deviated from being completely parallel to the axon and, with its processes, surrounded the soma and proximal dendrites of NF-200+RGCs (Figure 9A2,B2,C2). In the retinal-like section effect produced by the pressure exerted by the cover slip on the whole-mount, we observed that rod-microglia processes were related to the dendrites of NF-200+RGCs in the IPL (Figure 5B1-B3).

### 2: Scleral group

**Figure 10:**
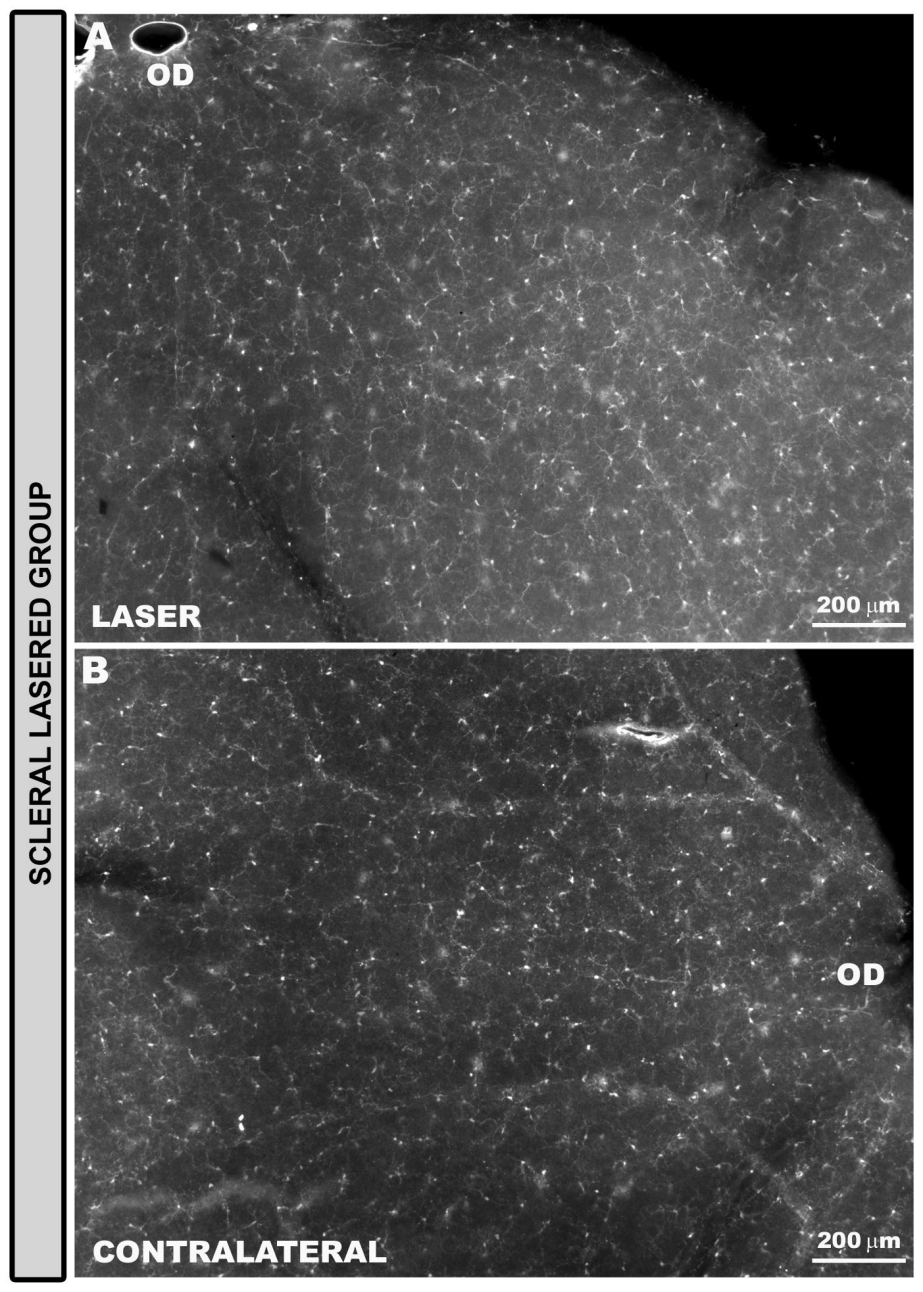
Rod-like microglia were absent in the scleral lasered group of eyes. Iba-1+ cells in the NFL and RGC layer. Retinal whole-mounts. In eyes receiving laser in the non-draining portion of the sclera, lasered (A) as well as contralateral untreated eyes (B) Iba-1+ cells were distributed in a mosaic of tiled cells that built networks throughout the entire retina. No rod-like microglia was detected in the NFL of this group of animals (NFL: nerve-fibre layer; RGC: retinal ganglion-cell layer; OD: optic disc).

**Figure 11:**
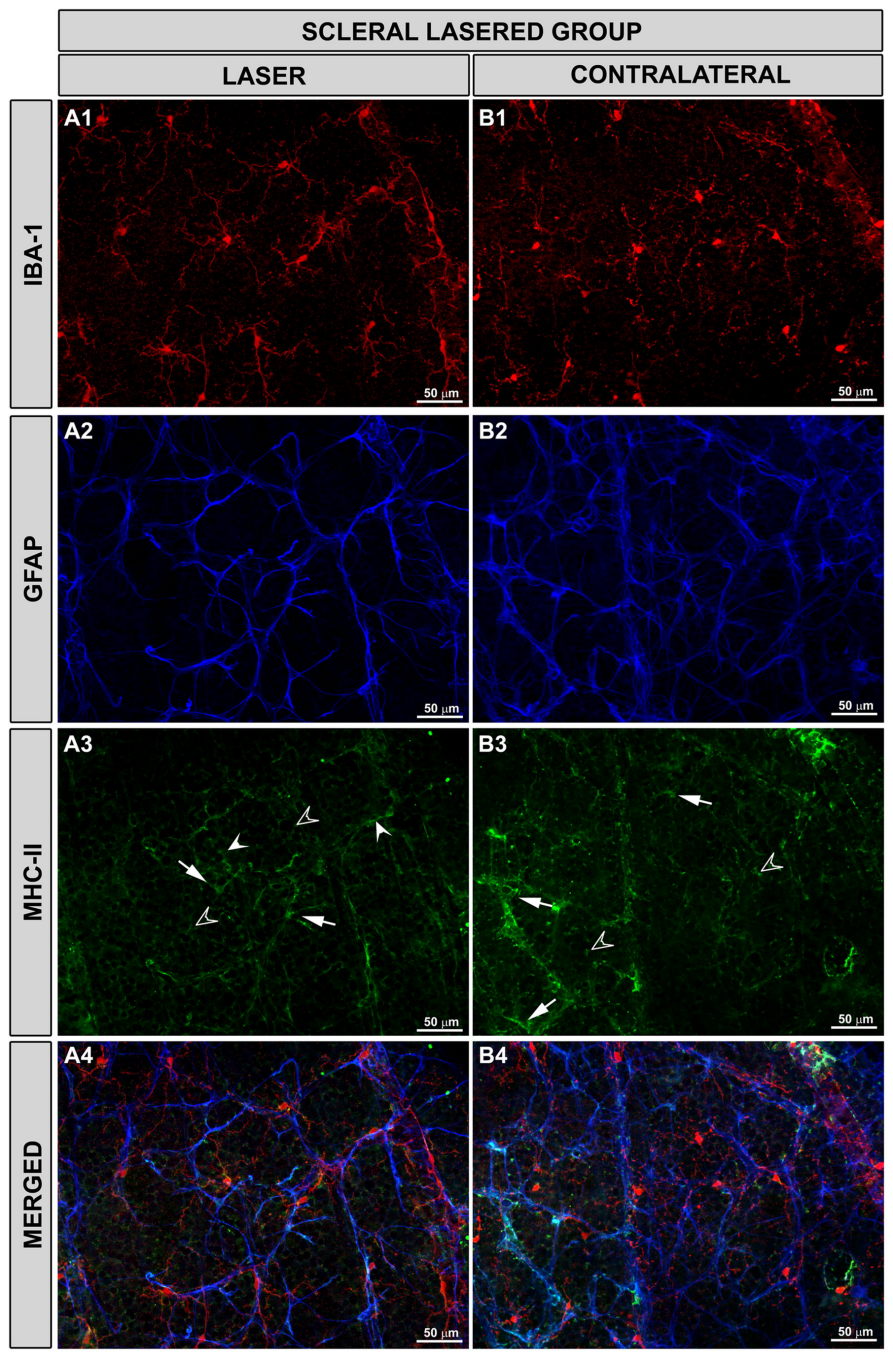
Macroglial MHC-II expression was upregulated in the scleral lasered group of eyes. Iba-1, GFAP and MHC-II immunostaining. Retinal whole-mounts. Both in lasered (A1-A4) and contralateral untreated (B1-B4) eyes, MHC-II expression was upregulated in astrocytes (arrow) and Müller cells (empty arrowhead). Some microglia in the NFL (arrowhead) had a constitutive MHC-II expression. (NFL: nerve-fibre layer).

 The IOP values of eyes that received laser in the sclera (15.69±0.12 mmHg) did not present significant differences with their contralateral (15.94±0.02 mmHg) eyes and naïve eyes (16.16±3.11 mmHg). Similarly to naïve lasered as well as contralateral eyes in the scleral group had ramified Iba-1+ cells in the NFL distributed in a mosaic of tiled cells that built networks throughout the entire retina (Figure 10) and exhibited a weak constitutive MHC-II expression (Figure 11A3). No rod-like microglia were detected in the NFL of this group of animals (Figures 10, 11A1,B1).

MCH-II expression was upregulated in Müller cells and some astrocytes No differences in terms of MHC-II expression by the macroglia were found between treated and untreated fellow eyes (Figure 11A2-4,B2-4). 

## Discussion

It has been reported that in the experimental model of OHT used in this work, 15 days after laser treatment the microglia of untreated as well as OHT-eyes had signs of activation [32]. However, two main issues remain unknown: i) whether microglial activation is secondary to laser injury or higher IOP; and ii) if microglial activation is IOP related, whether or not these activated cells could be exerting different functions contributing to damage or preservation of the RGC in the OHT-eyes and contralateral normotensive eyes, respectively. Therefore the present study was focused on evaluating the contribution of laser injury and IOP to the microglial activation observed in the NFL and on analysing the differential behaviour of activated microglial cells in this retinal layer after 15 days of lasering. 

In the model of laser-induced OHT used in the present work, a substantial increase in the IOP was evident 24 h after lasering limbal and episcleral veins. This continued for 4 days and then gradually returned to the basal value after the fifth day, so that by one week after lasering, the IOP values in the treated animals were comparable for both eyes [33]. 

No morphological signs of activation were detected in the retinal microglia of those animals receiving laser in the non-draining portion of the sclera (the scleral group), in which the IOP values did not differ from control. 

With respect MHC-II expression by the glial cells of the retina, differences appeared 15 days after lasering, depending on the location of laser application. Only eyes in the OHT group (treated eyes as well as fellow untreated eyes) had an upregulation of the MHC-II expression by the retinal microglia. With respect the macroglia, MCH-II was upregulated in both groups of animals receiving laser (OHT and scleral), although there was a difference between them: in the scleral group, where IOP values not differ significantly from naïve eyes, the upregulation of MHC-II expression by the macroglia was similar in treated eyes and untreated fellow eyes. On the contrary, in the OHT group the upregulation of MCH-II expression was observed mainly in Müller cells in OHT-eyes and mainly in astrocytes in their fellow normotensive eyes [32]. In view of these results, although the laser injury in the anterior segment of the eye induced some effects on the retinal macroglia, it seems that in this model of laser-induced OHT microglial activation is at least in partly related to the rise in IOP. 

It has been reported that in mouse retina, cannulation of the anterior chamber, with and without associated acute elevation of the IOP, trigger MHC-II upregulation in perivascular macrophages and vitreal hyalocytes. This result was not detected in the contralateral fellow eyes 1 week after the rise in IOP [41]. These contradictory findings in comparison with our results could be due to the different mechanisms involved in varying experimental models of elevated IOP.

A reactive microgliosis secondary to neurodegeneration has been reported in axotomy, ischaemia, and acute ocular hypertension [42-51]. In the present study the comparison between OHT-eyes and their contralateral ones revealed that in both eyes, Iba-1+ cells showed signs of activation and upregulation of the MHC-II expression. However, a relevant difference between treated and untreated eyes was that only OHT-eyes had Iba-1+ cells with rod-like morphology. In addition, rod-like microglial cells were not found in the scleral group. The fact that rod-like microglia was restricted to eyes with OHT lead us to postulate that this cell phenotype is not associated to the laser injury.

Rod-like microglial cells were related to axons but not to retinal blood vessels. Notably, they were apparently more evident in the central than in the intermediate zone of the retina and were scarcely seen in the retinal periphery, where the concentration of axons is reduced [52-54].

It is well known that in the brain rod-like microglia is related to neurodegeneration [55,56]. Accordingly, in this experimental model, the presence of rod-like microglia was restricted to eyes with OHT in which neuronal damage is present [33] but absent in the contralateral untreated eyes where macro- and microglia showed signs of activation but neurodegeneration appears to be absent [32,33]. This is the first available report of such an observation in ocular hypertension.

The presence of rod-like microglia has been associated mainly with axonal injury [22]. It bears noting that, in the present study, rod-like microglia were related to axons and were in apparent contact with the soma and dendrites of RGCs exhibiting features of degeneration (accumulation of phosphorylated neurofilaments revealed by the NF-200+ staining). A striking feature of this relationship was that a rod-microglia deviated from its straight parallel location to the axon and, with its processes, surrounded the soma and dendrites of NF-200+RGCs. The relationship between rod-like microglia and dendritic degeneration have been reported in the brain during ischaemia [57], after injury to the hypoglossal nerve [58] and during inflammation in the cortex [59]. It seems that the rod-like microglia might be involved in the active removal or “stripping” of these synaptic contacts [21,25]. The fact that rod-like microglia in the present study were positive for CD68, a marker associated to clearance of cells debris [60], could support this statement. Rod-like microglia could also participate in the remodelling of synaptic circuitry disrupted, possibly by tagging some synapses for elimination during prolonged direct contact after ischaemic insult [61]. This capacity could be favoured by the migratory capability of microglia towards vulnerable areas, which could contribute to circuits reorganization clearing degenerating synapses [23,62]. 

Although MHC-II upregulation in rod-like microglia could make these cells work as potential immunocompetent antigen-presenting cells, the fact that these cells did not express the co-stimulatory molecule CD86 could be contribute to downregulating of the immune response [63,64]. 

Ym1 is a marker expressed by alternatively activated macrophages associated to recovery and function restoration [65,66]. Ym1 is activated transiently in peripheral macrophages and in the CNS, suggesting that it may be involved in the down-regulation of inflammation by competing leukocyte trafficking for binding sites on local extracellular matrix [67]. According to what has been reported in the brain ischaemic injury in mice [60], this transient activation could be the reason for observing Ym1 positivity in only a few globular cells 15 days after unilateral laser-induced OHT. 

Some of the characteristics of the rod-like microglia observed herein, such as morphological features, their relationship with axons but not with blood vessels, or their expression of known activation markers (MHC-II, CD68) coincide with those reported under many neuropathological and experimental conditions including stroke, Alzheimer’s disease, and encephalitis [22]. It has been proposed that the preserved neuronal tissue after a diffuse insult is critical for the formation of rod-like microglial cells [12,13,68]; with extensive cellular damage or necrosis, only phagocytic microglia can be found.

The capacity of the microglia to adopt a bidirectional orientation from progressing to regressing stages has been recently reported. Using histological criteria, this process has been divided into 6 stages of activation and deactivation [69]. According to this classification, rod-like microglia in the NFL of OHT-eyes would be in phase 5A, which corresponds to Iba-1+ and CD68+ cells. In addition, more advanced stages were also observed in our study. These were characterized by more intense CD68 immunostaining, shorter processes, and rounder somas. 

With respect to the relationships that rod-like microglia establish between each other when forming trains, it is worth underlining what we described these as soma-soma, which consisted of two somas of neighbouring cells in apparently close contact. This situation could correspond to microglial cells in division, as revealed by BrdU studies in which both nuclei of the cells in contact were labelled [69,70]. It has been reported that at more advanced stages of activation the presence of cells with multiple nuclei could be the result of microglial cells fusing with each other [69]. The clustering reported by others [71] could be a precursor stage for the eventual fusing of the cells [69]. It has been suggested that cytokines and other factors induce multinucleated giant cells [72] and regulate morphological, as well as functional, changes to all three forms of microglia; activated ameboid, proliferating rod-shaped, and ramified resting microglia [70].

Although laser treatment induced changes in the retina of contralateral eyes independently of the location of laser application, the characteristic of this reaction varied depending on whether or not there was a rise in IOP in the treated eye. In all instances, laser treatment induced a macroglial reaction but only in eyes contralateral to OHT was the microglia involved. 

There are other instances of contralateral effects after unilateral eye injury [30,73,74]. It has been suggested that multiple cell responses in the contralateral eye could be due to the crossing fibres at the optic chiasma or some retino-retinal fibres present in rodents [30]. The fact that the microglia showed an immediate and clear pattern of activation within the contralateral ON and retina, lead some authors suggest haematic involvement in transferring information to the contralateral eye [30]. Among the possible mechanisms participating in the glial changes observed in the contralateral normotensive eye is the immune response [75]. Immune-related data in patients with glaucoma [76] and the capacity of the optic nerve glia to express HLA-DR molecules [73] support this hypothesis. Another potential explanation would be that changes in the contralateral eye are part of a general activation due to the sensitivity of microglia to any inflicted injury to the nervous system [74]. It is now well accepted that neurogenic mechanisms contribute to the symmetrical spread of inflammation in rheumatoid arthritis [77,78] and that transneuronal signalling between damaged neurons and their contralateral homologues prevent the spread of peripheral nerve damage [79]. It has been recently reported that retinal laser burns to one eye abrogated ACAID bilaterally [80]. The authors postulate that substance P transmits early inflammatory signals from the lasered retinal to the contralateral eye to induce changes to ocular immune privilege and has a central role in the bilateral loss of ACAID [81]. Whether some of the mentioned mechanisms are involved in the changes noted in the contralateral untreated eyes in our study deserves further investigation.

Our data suggest that 15 days after lasering, microglia activation in the retina is not related to laser injury but rather to IOP-derived effects. The presence of rod-like microglia was restricted to OHT. In contralateral eyes to OHT, despite the presence of signs of activation in microglia as well as macroglia (morphological changes and MHCII upregulation), neither rod-like microglia nor degenerated NF-200+RGCs were observed. Thus, it appears that the neurodegeneration associated to OHT induce the rod-like microglia phenotype. 

The present study describes the differential behaviour of activated microglia that may accompany OHT-induced injury. The observations made support the role of the immune system in the pathogenesis of glaucomatous neurodegeneration. Whether or not the observations reported here could appear in other models of experimental OHT deserves further investigation. 
